# Antioxidant and antimicrobial potential of water lily extracts and their effects on the quality of frozen Nile tilapia (*Oreochromis niloticus*) fillets

**DOI:** 10.1002/fsn3.3084

**Published:** 2022-10-01

**Authors:** Md. Apon Dulal, Israt Jahan, Md. Golam Rasul, Md. Rabiul Islam, Murshida Khan, A. K. M. Azad Shah

**Affiliations:** ^1^ Department of Fisheries Technology Bangabandhu Sheikh Mujibur Rahman Agricultural University Gazipur Bangladesh; ^2^ Department of Aquaculture Bangabandhu Sheikh Mujibur Rahman Agricultural University Gazipur Bangladesh

**Keywords:** antimicrobial, antioxidant, Nile tilapia, quality, sensory evaluation, shelf life

## Abstract

This study was investigated to evaluate the antioxidant and antimicrobial potential of water lily extracts and their effects on the quality of Nile tilapia (*Oreochromis niloticus*) fillets during frozen storage (−18 ± 1°C). Antioxidant and antimicrobial activities of water lily extracts, and chemical, microbiological, and sensory qualities of fish fillets were assessed. Results showed that the highest total phenolic content (34.07 mg GAE/g) and total flavonoid content (32.67 mg QE/g extract) were found in the ethanolic extract and the lowest in water extract of water lily. The ethanolic extracts of water lily also exhibited the highest antioxidant capacities and antimicrobial activities than other hydroethanolic and water extracts. The water lily extracts‐treated fish fillets showed the highest potentiality in lowering the pH, total volatile basic nitrogen, and thiobarbituric acid reactive substances than the untreated fillets throughout the storage period. Moreover, ethanolic extracts of water lily exhibited comparatively higher efficacy in inhibiting bacterial growth in fish fillets than other extracts‐treated fillets. The ethanolic extracts‐treated fillets also showed better sensory attributes than hydroethanolic and control fillets. Therefore, ethanolic extract of water lily can be used as a natural preservative in enhancing the quality and prolonging the shelf life of Nile tilapia fillets during frozen storage.

## Graphical abstract

Extension of shelf life using natural antioxidant like water lily extracts is desirable for highly perishable seafood preservation than the synthetic one. In this study, water lily ethanolic extract has considerable amount of antioxidant and antimicrobial potentiality, which effectively reduced the quality deterioration, inhibited the lipid oxidation, slowed down the microbial growth and improved the overall sensory attributes in frozen Nile tilapia fillets. Based on the chemical microbiological and sensory data, it is suggested to use ethanolic extracts for the enhancement of quality and shelf life of Nile tilapia fillets during frozen storage. This preservation strategy can be applicable in fish/seafood processing industry and in household purpose as well.

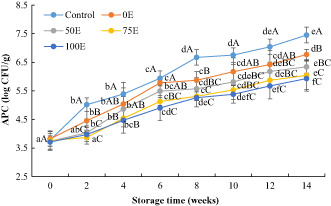

## 1 INTRODUCTION

Bangladesh is one of the leading fish‐producing countries in the world and the fisheries sector is one of the most productive and dynamic industries, which has a tremendous potential for future development in the agro‐based economy of Bangladesh. In 2019–2020, the total fish production of Bangladesh was 4.503 million metric tons (MT); among them, Nile tilapia (*Oreochromis niloticus*) production was 371,263 MT (Department of Fisheries (DoF), 2020). Tilapia is one of the most suitable fish species for intensive farming and trade in Bangladesh. Several species of tilapia are cultured commercially, among them, Nile tilapia is preferred by the people due to its white meat, firm texture, delicate taste, easy filleting, no “Y”‐shaped bones, high growth rate, and adaptability to different conditions (Jory et al., 2000). Conversely, fish are recognized as highly perishable food items, having a relatively short shelf life because of many inherent elements such as neutral pH, low connective tissue, high water‐holding capacity, muscle enzymes, and natural microbial flora (Kilincceker et al., 2009).

To prevent chemical deterioration and delay microbial growth in fish, various preservation methods are used. Among them lowering the temperature of fish and use of chemical preservatives such as nitrites, sulfites, ethylene diamine tetra acetic acid (EDTA), lactic acid, benzoic acid, and ascorbic acid are commonly practiced (Ghaly et al., 2010), but they do not effectively inhibit the quality deterioration of fish. Moreover, excessive use of chemical additives has proved to be carcinogenic, causing health problems such as cancer and birth defects (Maqsood et al., 2014). In contrast, it is generally accepted that natural antioxidants are more potent, efficient, and safer than synthetic ones (Tavasalkar et al., 2012). Therefore, it is important to replace synthetic antioxidants with natural preservatives having both antioxidant and antibacterial activities that prolong the shelf life of fish and fishery products.

Plants are revealed to be a rich source of natural antioxidant and antimicrobial compounds (Pezeshk et al., 2016), and plant‐based antioxidants are preferred for their multiple modes of action and are nontoxic in nature. The water lily (*Nymphaea nouchali*), locally known as “Shapla,” is an aquatic plant that grows abundantly in almost all shallow natural waterbodies and has been designated as the national flower of Bangladesh. The leaves, flowers, and stems contain various bioactive compounds such as polyphenols and flavonoids and have been found to have antioxidant, antimicrobial, antidiabetics, and hemolytic properties (Alam et al., 2017; Jahan et al., 2012). Various secondary metabolites like sterols, alkaloids, saponins, tannins, and flavonoids have been isolated from this plant and have shown antibacterial activities (Parimala & Shoba, 2013). In addition, these compounds can prevent oxidative modification by neutralizing free radicals, oxygen scavenging, or decomposing peroxides through their antioxidant activities (Jahan et al., 2012). Many studies have shown the preservative effect of plant extracts on seafood such as crucian carp (Li et al., 2012), yellow croaker (Zhao et al., 2013), Nile tilapia (Jadhav & Anal, 2018; Khalafalla et al., 2015), and *Pangasius* fillet (Deepitha et al., 2021). It has been reported that 2% pomegranate seed extract effectively retards the spoilage rate and extends the shelf life of chub mackerel mince during frozen (−18°C) storage (Ozgen et al., 2011). Besides, lotus (*Nelumbo nucifera*) petals extract improves the quality of yogurt during fermentation (Chen et al., 2021). However, to the best of our knowledge, no studies have so far been reported concerning the antioxidant and antimicrobial potential of ethanolic/hydroethanolic extracts of water lily and their application in Nile tilapia preservation. Therefore, this study aimed to evaluate the antioxidant and antimicrobial activities of water lily extracts and their effects on the quality and shelf‐life extension of Nile tilapia fillets during frozen storage.

## 2 MATERIALS AND METHODS

### 2.1 Materials and chemicals

A total of 30 live Nile tilapia (*Oreochromis niloticus*) with an average weight of 900 ± 150 g were collected from a fish farm located at Kapashia, Gazipur district, Bangladesh, and slaughtered by immersion in ice‐cold water (hypothermia). The fish were kept immediately in an insulated box maintaining fish:ice ratio of 1:2 and transported to the Fish Processing Laboratory, Department of Fisheries Technology of Bangabandhu Sheikh Mujibur Rahman Agricultural University (BSMRAU), Gazipur.

Water lily (*Nymphaea nouchali*) was collected from waterbodies located in Gazipur district, Bangladesh, and transported to the laboratory. Obtained water lily (leaves, flowers, and stems; aged about 3 months) was washed thoroughly with potable water, kept under shade, and dried at room temperature (28–30°C) for 7 days. The dried water lily was cut into small pieces and powdered using a grinder. The water lily powder was packed in plastic bags and stored at −18°C until analysis. All the chemicals and solvents used in this study were of analytical or HPLC grade.

### 2.2 Preparation of water lily extracts

Powdered water lily (10 g) was drenched separately in various solvents such as 0E (0% ethanol or distilled water), 50E (50% ethanol), 75E (75% ethanol), and 100E (100% ethanol or absolute ethanol), and gently mixed by shaking and extracted for 72 h at room temperature (28–30°C). The crude extracts were filtered, and the residue was reextracted in the same solvents for 72 h. The filtered crude extracts obtained were pooled and concentrated using a rotary evaporator (Stuart, Stone, Staffordshire, UK). The crude extracts obtained were weighed to determine extraction yield and stored in the dark at 4°C for further use. Stock solutions of water lily extracts were prepared for analysis by dissolving 1000 μg of dried water lily extract in 1 ml of methanol.

### 2.3 Phytochemical content and antioxidant assays

The total phenolic content (TPC) of water lily extracts was determined by Folin–Ciocalteu's phenol method (Taga et al., 1984), and the values were expressed as mg gallic acid equivalent per gm (mg GAE/g). The total flavonoid content (TFC) was measured following the method described by Zishen et al. (1999) and results were expressed as mg quercetin equivalent (QE)/g of extract. The DPPH (1,1‐diphenyl‐1‐picrylhydrazyl) radical scavenging activity of crude extracts was measured following the method of Yen and Chen (1995). The EC_50_ value was expressed as a sample concentration that can quench 50% of DPPH free radicals. The ABTS (2,2′‐azino‐bis[3‐ethylbenzothiazoline‐6‐sulfonic acid] diammonium salt) radical scavenging activity was determined following the method of Re et al. (1999). The EC_50_ value was determined as a sample concentration that can quench 50% of ABTS radicals.

### 2.4 Antibacterial assay

The antibacterial activity of water lily extracts was carried out *in vitro* using agar well diffusion technique (Berghe & Vlietinek, 1991). Two food pathogenic bacteria such as *Escherichia coli* (ATCC 25922) and *Staphylococcus aureus* (ATCC 25923), and two food spoilage bacteria, *viz*. *Enterococcus faecalis* (ATCC 29212) and *Pseudomonas aeruginosa* (ATCC 27853), were used to determine antibacterial activity. Mueller Hinton (MH) agar plates were prepared, a sterile cotton swab was used to spread the subcultured bacterial strains from inoculated nutrient broth evenly on the agar plate surface from three directions, and plates were kept resting for the absorption of the bacterial inoculums. Three equidistant wells on each MH plate were made using a cork borer (6 mm), and the wells were loaded with 100 μl of water lily extracts at a concentration of 12.5, 25, and 50 mg/ml. Afterward, the plates were incubated at 37°C for 24 h. The antibacterial activity was indicated by measuring the inhibition zone around each well (Alshalmani et al., 2014). Ethanol was used as negative control and antibiotic gentamicin was used as a positive control to determine the efficiency of water lily extracts.

### 2.5 Preparation of fish fillet

The fish were gutted, washed, and filleted using sterilized sharp knives on cutting boards. After removal of the head and bone, two fillets with skin were obtained from each fish. Then, the fillets were cut into small pieces, and the average weight of each piece was 45.4 ± 14.1 g.

### 2.6 Coating application and storage

Fish fillets were randomly divided into five treatments, *viz*. control (distilled water or without water lily extracts), 0E (water extract solution), 50E (50% ethanolic extract solution), 75E (75% ethanolic extract solution), and 100E (100% ethanolic extract solution), each having three replicates. All the extracts were diluted into distilled water at a concentration of 2% (w/v) for coating application. The control treatment fillets were dipped in distilled water, while other treatments were immersed in water lily extract solution as described above. The duration of the dipping treatment was 10 min at 4°C. The fish fillets were air dried for 5 min in order to form an edible coating. All the samples were individually packed in airtight polyethylene zip bags and stored at −18 ± 1°C for 14 weeks. During storage, all the treated and untreated samples were analyzed periodically (every 2 weeks) to determine the overall quality of fish fillets.

### 2.7 Chemical analyses

The proximate composition (moisture, crude protein, crude lipid, and ash content) of fish fillets was determined following the standard procedure given by the Association of Official Analytical Chemists (AOAC, 2005). The pH value was directly measured using a pH meter according to the method of Afrin et al. (2021). The thiobarbituric acid reactive substance (TBARS) was measured following the method of Buege and Aust (1978) and the value was expressed as mg malondialdehyde (MDA)/kg of flesh. The total volatile basic nitrogen (TVB‐N) was determined according to the AOAC (2005) method, and the value was expressed as mg N/100 g of muscle.

### 2.8 Microbiological analysis

The aerobic plate count (APC) of fish fillets was determined following the method described by Maturin and Peeler (2001). The APC was determined using plate count agar (Hi Media) and expressed as log colony‐forming units per gram or log CFU/g of fish flesh.

### 2.9 Sensory evaluation

The sensory evaluation of fish fillets was performed following the method described by Ojagh et al. (2010). Raw fillets were assessed by eight trained assessors (ages between 23 and 39 years) from the Department of Fisheries Technology. Sensory characteristics were evaluated using a 5‐point scale with the following descriptive terms: color (1 = extreme discoloration to 5 = no discoloration), odor (1 = extremely undesirable/off‐odor to 5 = extremely acceptable), texture (1 = very soft to 5 = firm), and overall acceptability (1 = dislike extremely to 5 = like extremely) of the samples. A sensory score of less than 4 implies that the fish was rejected.

### 2.10 Statistical analyses

Statistical analyses were accomplished using the Statistical Analysis System (SAS, 2003, Version 9.1, SAS Institute). All experiments were performed in triplicate based on a completely randomized design. Data were subjected to a two‐way analysis of variance (ANOVA) followed by a post hoc test using Duncan's multiple‐range test to identify the significance of differences among the means at *p* < .05.

## 3 RESULTS AND DISCUSSION

### 3.1 Extract yield and phytochemical contents of water lily extracts

The yield of water lily extracts ranged from 17.11% to 31.27% (Table 1). Significantly (*p* < .05) the highest yield was observed in 0E (31.27%), while the lowest yield was found in 100E (17.11%). This suggests that the extract yield increases with the increase in solvent polarity, and generally, the plant material contains high levels of polar compounds that are soluble in solvents with high polarity. This result is consistent with the extract yield of *Limnophila aromatica* (Do et al., 2014) and some other medicinal plants (Kuppusamy et al., 2015). Phenolic compounds are parts of secondary metabolites that are mostly found in plant species with numerous structural compositions (Alara et al., 2021). In this study, the TPC of water lily extracts ranged from 28.93 mg GAE/g to 34.07 mg GAE/g. Significantly (*p* < .05), the highest TPC was found in 100E (34.07 mg GAE/g) followed by 75E (33.32 mg GAE/g), 50E (31.12 mg GAE/g), and 0E (28.93 mg GAE/g), which suggests that TPC decreased with the increasing solvent polarity. Nagavani and Rao (2010) also found that the TPC was higher in ethanolic extract (24.0 mg GAE/g) than in aqueous extract (6.13 mg GAE/g) in dry flowers of *N. nouchali*. Similarly, the significantly highest TFC was found in 100E (32.67 mg QE/g extract) and the lowest in 0E (16.15 mg QE/g) (Table 1). These results are in agreement with Do et al. (2014), who observed that 100% ethanolic extracts of *L. aromatic* showed the highest TFC (31.11 mg QE/g) than water extract (4.04 mg QE/g). Kahkonen et al. (1999) also stated that flavonoids are probably the most important natural phenolics due to their broad spectrum of chemical and biological activities and this might be the fact that the TFC was higher in ethanolic extracts.

**TABLE 1:** Extract yield and phytochemical content of water lily extracts

Extract	Extract yield (g/100 g of dry weight)	Total phenolic content (mg GAE/g)	Total flavonoid content (mg QE/g)
0E	31.27 ± 1.05^a^	28.93 ± 0.21^d^	16.15 ± 0.27^d^
50E	27.19 ± 0.87^b^	31.12 ± 0.21^c^	22.36 ± 0.38^c^
75E	22.37 ± 0.95^c^	33.32 ± 0.05^b^	26.09 ± 0.35^b^
100E	17.11 ± 1.21^d^	34.07 ± 0.13^a^	32.67 ± 0.33^a^

*Note*: The values represent means of triplicates ± SD. ^a–d^Means with different letters in each column indicate significant differences (*p* < 0.05).

Abbreviations: 0E, 0% ethanol extract (water extract); 100E, 100% ethanol extract; 50E, 50% ethanol extract; 75E, 75% ethanol extract.

### 3.2 Antioxidant capacity of water lily extracts

The DPPH and ABTS radical scavenging activities of water lily extracts in different solvents are shown in Table 2. The DPPH has been used extensively to investigate the free radical scavenging activities of compounds (Duan et al., 2006). The lower EC_50_ value regards the higher antioxidant activity. In this study, the EC_50_ values of water lily extracts ranged from 30.15 to 51.85 μg/ml. Significantly (*p* < 0.05) the highest EC_50_ value was found in 0E (51.85 μg/ml), followed by 50E (47.35 μg/ml), 75E (33.33 μg/ml), and 100E (30.15 μg/ml). Similarly, the highest ABTS value (EC_50_) was observed in 0E (18.09 μg/ml), while 100E showed the lowest EC_50_ value (13.65 μg/ml) (Table 2). It has been reported that DPPH radical scavenging activities were significantly correlated with the total abundance of phenolic compounds (Tayade et al., 2013). The results of this study are comparable with Madhusudhanan et al. (2011), who found that the ethanolic extract of *N. alba* flower showed the highest inhibition of DPPH activity than the aqueous extract. Moreover, Daffodil and Mohan (2014) explained that the most polar solvent extract showed a maximum scavenging activity than low polar solvent extracts. It has been reported that ethanolic extract of *P. tetrastromatica* exhibited higher antioxidant and antimicrobial activity due to the presence of total phenolic content (Layana et al., 2019).

**TABLE 2:** Antioxidant capacity of water lily extracts

Extract	DPPH radical scavenging activity (EC_50_ [μg/ml])*	ABTS radical scavenging activity (EC_50_ [μg/ml])
0E	51.85 ± 0.22^a^	18.09 ± 0.26^a^
50E	47.35 ± 1.27^b^	15.90 ± 0.12^b^
75E	33.33 ± 1.01^c^	14.55 ± 0.19^c^
100E	30.15 ± 1.01^d^	13.65 ± 0.05^d^

*Note*: The values represent means of triplicates ± SD. ^a–d^Means with different letters in each column indicate significant differences (*p* < 0.05).

Abbreviations: 0E, 0% ethanol extract (water extract); 100E, 100% ethanol extract; 50E, 50% ethanol extract; 75E, 75% ethanol extract.

* EC_50_ means the half maximal effective concentration.

### 3.3 Antibacterial activity of water lily extracts

*In vitro* antibacterial activity of water lily extracts against four bacterial strains (*E. coli, S. aureus, E. faecalis, and P. aeruginosa*) is shown in Table 3. Results showed that 100E exhibited the highest zone of inhibition against all the bacterial strains followed by 75E, 50E, and 0E, which suggest that antibacterial activity might be decreased with the increase in solvent polarity (Singtongrat et al., 2012). Ethanolic extract (100E) showed comparatively higher antibacterial activity (+++) at a concentration of 50 mg/ml against all the bacterial strains than other concentrations, which was comparable with the inhibitory activity of gentamicin. On the other hand, water extracts showed the lowest antibacterial activity at different concentrations. The antibacterial results of this study can be presented in the following order, 100E > 75E > 50E > 0E for solvents, where larger inhibition zones were found according to the following order, 50 > 25 > 12.5 mg/ml of concentration. Results of this study showed that ethanol was the most suitable solvent for the extraction of phenolic and flavonoid compounds because of its solvent polarity (Mickymaray, 2019). The water lily is a rich source of astragalin, corilagin, catechin, epicatechin, gallic acid, isokaempferide, kaempferol, quercetin‐3‐methyl ether, and quercetin that have shown potent antioxidant and antimicrobial activities (Alam et al., 2017). Moreover, the effectiveness of phenolic and flavonoid compounds against some microorganisms might be due to their one carbonyl group structures produced by a plant that responds to microbial infection (Dixon et al., 1983). Akinjogunla et al. (2010) also reported that *N. lotus* leaf extract exhibited antibacterial activity against the growth of *S. aureus, Bacillus cereus*, MRSA, and *P. aeruginosa*.

**TABLE 3:** Antibacterial activity of water lily extracts in different solvents

Bacterial strain	Concentration (mg/ml)	Extraction solvents			
		0E	50E	75E	100E
*Staphylococcus aureus*	12.5	+++	+++	+++	+++
	25	+++	+++	+++	+++
	50	+++	+++	+++	+++
	Gentamicin	+++	+++	+++	+++
*Escherichia coli*	12.5	−	+	+	++
	25	+	++	++	++
	50	+	++	++	+++
	Gentamicin	+++	+++	+++	+++
*Enterococcus faecalis*	12.5	−	+	+	++
	25	++	++	++	++
	50	++	++	+++	+++
	Gentamicin	++	++	++	++
*Pseudomonas aeruginosa*	12.5	+	++	+	++
	25	+	++	++	++
	50	++	++	++	+++
	Gentamicin	+++	+++	+++	+++

*Note*: Inhibition zone 7 mm or <7 mm is referred to as inactive (−); >7 mm and <9 mm is interpreted as trace active (+); between 10 mm and 14 mm is interpreted as moderately active (++); and >14 mm is interpreted as highly active (+++) (Alshalmani et al., 2014). 0E: 0% ethanol extract (water extract), 50E: 50% ethanol extract, 75E: 75% ethanol extract, and 100E: 100% ethanol extract.

### 3.4 Proximate composition

The moisture, crude protein, crude lipid, and ash contents of Nile tilapia fillet was 77.21%, 15.32%, 5.33%, and 2.12%, respectively, on a fresh matter basis. Many studies have reported on the proximate composition of Nile tilapia from various locations, and the results of this study were more or less similar to those studies (Alsaggaf et al., 2017; Jadhav & Anal, 2018). The variations in the proximate composition of fish flesh can be correlated with their nutrition, size of fish, habitat, feeding habit, time of catching, gender, spawning cycle, and other environmental factors.

### 3.5 Changes in pH values

**FIGURE 1:**
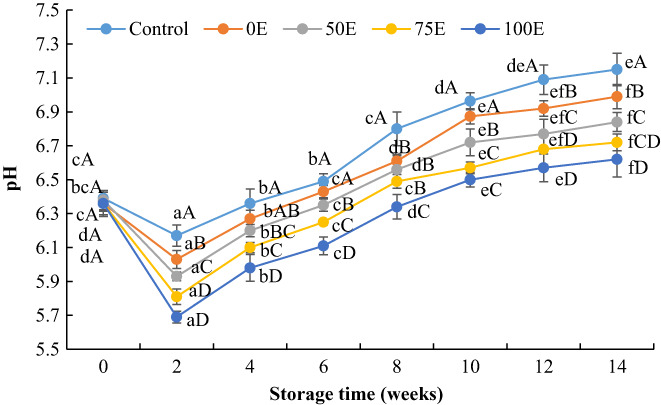
Changes in pH values of Nile tilapia fillets during frozen storage. Results represent means ± SD in triplicate. ^a–f^ Small letters in each line indicate significant (*p* < 0.05) differences of means within the storage time. ^A‐D^ Capital letters indicate significant (*p* < 0.05) differences of means within the treatments.

Changes in pH values of Nile tilapia fillets during frozen storage are shown in Figure 1. The initial pH of fresh Nile tilapia fillet ranged from 6.36 to 6.39, which indicates that the fillet was fresh before frozen storage (Li et al., 2012). The pH values of Nile tilapia fillets were initially decreased within 2 weeks of storage and then the values were increased gradually up to 14 weeks of storage. The initial reduction of pH in Nile tilapia fillet could be explained by the accumulation of lactic acid resulting from glycogen consumption in fish muscle (Cai et al., 2014). After 14 weeks of frozen storage, the pH of the control fillet was 7.15, whereas the pH of fillets treated with 0E, 50E, 75E, and 100E were 6.99, 6.84, 6.72, and 6.62, respectively. The pH values of water lily extracts‐treated fillets were significantly (*p* < .05) lower than that of the control. The subsequent increase in pH value might be due to the formation of volatile basic nitrogenous compounds, such as trimethylamine and ammonia, that resulted from either microbial or endogenous enzymatic activities (Duman & Ozpolat, 2015).

### 3.6 Changes in thiobarbituric acid reactive substances (TBARS)

**FIGURE 2:**
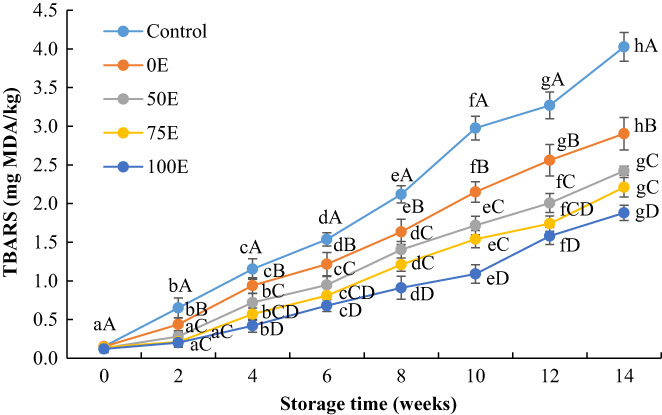
Changes in thiobarbituric acid reactive substances (TBARS) values of Nile tilapia fillets during frozen storage. Results represent means ± SD in triplicate. ^a‐h^ Small letters in each line indicate significant (*p* < 0.05) differences of means within the storage time. ^A‐D^ Capital letters indicate significant (*p* < 0.05) differences of means within the treatments.

The TBARS values of Nile tilapia fillets were 4.03 mg MDA/kg, 2.90 mg MDA/kg, 2.42 mg MDA/kg, 2.21 mg MDA/kg, and 1.88 mg MDA/kg for control, 0E, 50E, 75E, and 100E, respectively, after 14 weeks of storage (Figure 2). This indicates that the control fillets exceeded the acceptable range ((<2 mg MDA/kg for fresh fish) (Connell, 1990)) after 6 weeks of storage, however, 75E‐ and 100E‐treated fillets were still in acceptable range after 12 weeks and 14 weeks of storage, respectively. Moreover, a significant (*p* < .05) difference was found in the TBARS values during the subsequent frozen storage period, however, no significant (*p* > .05) difference was observed between the treatments of 50E and 75E over the storage period. Results of this study suggest that phenolic compounds present in the water lily extract might reduce the formation of free radicals in Nile tilapia fillets during frozen storage. It has been reported that pomegranate seed extract (2%) effectively reduces the formation of lipid hydroperoxides and TBARS values in chub mackerel mince during frozen (−18°C) storage (Ozgen et al., 2011).

### 3.7 Changes in total volatile basic nitrogen (TVB‐N)

**FIGURE 3:**
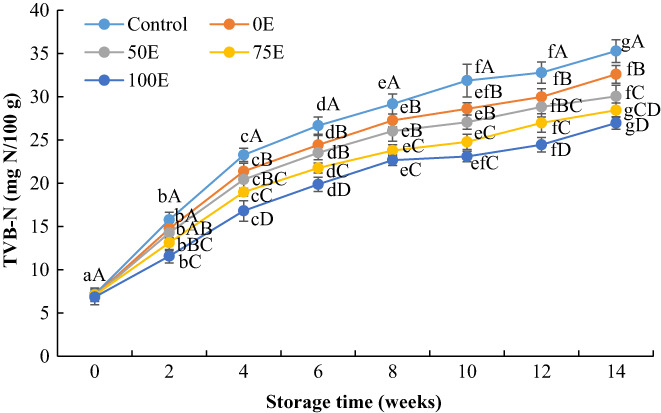
Changes in total volatile basic nitrogen (TVB‐N) values of Nile tilapia fillets during frozen storage. Results represent means ± SD in triplicate. ^a‐g^ Small letters in each line indicate significant (*p* < 0.05) differences of means within the storage time. ^A‐D^ Capital letters indicate significant (*p* < 0.05) differences of means within the treatments.

The TVB‐N values of Nile tilapia fillets increased gradually with the increase in storage time (Figure 3). After 14 weeks of storage, the TVB‐N values of Nile tilapia fillets reached 35.28, 32.60, 29.66, 28.44, and 26.96 mg N/100 g for control, 0E‐, 50E‐, 75E‐, and 100E‐treated fillets, respectively. The TVB‐N values of control and 0E extracts‐treated fillets exceeded the acceptable limit (<30 mg N/100 for fresh fish [Ocano‐Higuera et al., 2011]) after 4 and 6 weeks of storage, respectively, while the fillets treated with 50E, 75E, and 100E extracts exceeded the acceptable limit after 6, 10, and 12 weeks of storage, respectively. Comparatively lower TVB‐N values were found in water lily extracts‐treated fillets, which indicated that the extracts retarded the formation of alkaline compounds (Ojagh et al., 2010). Similar results were also observed by Khalafalla et al. (2015), who reported that the application of *Rosmarinus officinalis* and *Thymus vulgaris* extracts significantly reduced the formation of TVB‐N in Nile tilapia fillets during refrigerated storage. Elhafez et al. (2020) also reported that TVB‐N values of rosemary and thyme oil‐treated Nile tilapia fillets were significantly lower than untreated fillets during storage in refrigerated conditions.

### 3.8 Changes in aerobic plate count

**FIGURE 4:**
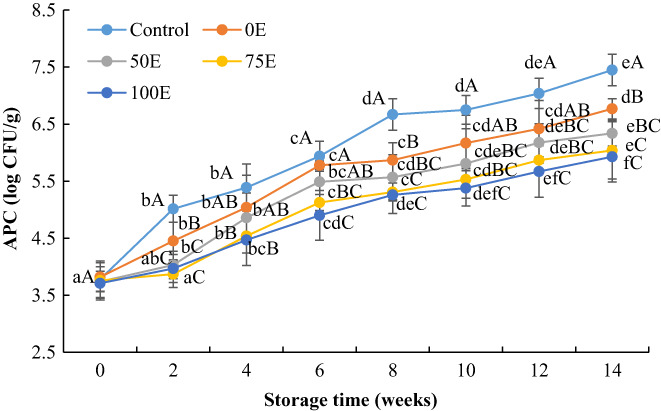
Changes in aerobic plate count (APC) values of Nile tilapia fillets during frozen storage. Results represent means ± SD in triplicate. ^a‐f^ Small letters in each line indicate significant (*p* < 0.05) differences of means within the storage time. ^A‐C^ Capital letters indicate significant (*p* < 0.05) differences of means within the treatments.

The initial APC of fresh Nile tilapia fillets ranged from 3.78 log CFU/g to 3.81 log CFU/g (Figure 4). After 14 weeks of frozen storage, the APC of control, 0E‐, 50E‐, 75E‐, and 100E‐treated fillets were 7.45 log CFU/g, 6.77 log CFU/g, 6.34 log CFU/g, 6.04 log CFU/g, and 5.93 log CFU/g, respectively, and the APC increased significantly (*p* < 0.05) throughout the storage period. The control fillet attained an APC value of 7.04 log CFU/g after 12 weeks of storage, which exceeded the microbiological acceptability limit of 7 log CFU/g for raw fish (Ojagh et al., 2010). However, 0E‐, 50E‐, 75E‐, and 100E‐treated fish fillets were still in the acceptable range after 14 weeks of storage. Results showed that all the water lily extracts‐treated fillets retard the bacterial growth during the storage period, which is more or less similar to the findings of Ozogul et al. (2011), who registered that a combination of rosemary and sage tea extract reduces the bacterial growth on sardine fillets during frozen storage. However, ethanolic extracts (100E) exhibited higher efficacy in inhibiting the bacterial growth and spoilage process in Nile tilapia fillets. These results suggest that the presence of phenolic compounds in the water lily extracts might be a reason for the reduction in APC in the treated fillets. It has been reported that the APC of *Tor khudree* was 4.05 log CFU/g at the end of frozen storage for 6 months when treated with beetroot peel extract (Maqbool et al., 2021).

### 3.9 Sensory evaluation

The sensory evaluation of Nile tilapia fillets during frozen storage is depicted in Table 4. All the sensory attributes (texture, color, odor, and overall acceptability) of control and 0E‐treated fillets were found to have unacceptable scores after 6 and 8 weeks of storage, respectively. The fillets treated with 75E and 100E extracts were found to have an acceptable score (above 4.0) up to 10 and 12 weeks of storage, respectively. The control fillets were spoiled after 6 weeks of storage because of a higher degree of lipid oxidation and bacterial growth. However, ethanolic extracts (100E)‐treated fillets showed better sensory attributes than control and water lily extracts‐treated fillets, which is more or less similar to Hassanin and El‐Daly (2013), who found that 3% garlic and 0.6% propolis extracts exhibited better sensory qualities than the control and other treatments during frozen storage (−18 ± 1°C) tilapia fillets. Moreover, control and beetroot peel extract‐treated *Tor khudree* steaks had acceptable sensory scores for about 5 months and 6 months, respectively (Maqbool et al., 2021).

**TABLE 4:** Changes in sensory characteristics of Nile tilapia fillets during frozen storage

Sensory attributes	Treatments	Storage time (weeks)							
		0	2	4	6	8	10	12	14
Texture	Control	5.00 ± 0.00_a_^a^	4.37 ± 0.15_a_^b^	4.12 ± 0.25_a_^b^	4.07 ± 0.14_a_^b^	3.56 ± 0.19_a_^c^	2.37 ± 0.08_a_^d^	1.26 ± 0.21_a_^e^	1.19 ± 0.22_a_^e^
	0E	5.00 ± 0.00_a_^a^	4.71 ± 0.21_ab_^ab^	4.63 ± 0.15_b_^ab^	4.44 ± 0.22_ab_^b^	4.24 ± 0.22_b_^bc^	3.84 ± 0.46_b_^c^	3.37 ± 0.41_b_^d^	2.98 ± 0.23_b_^d^
	50E	5.00 ± 0.00_a_^a^	4.79 ± 0.31_b_^ab^	4.68 ± 0.34_b_^ab^	4.45 ± 0.19_ab_^bc^	4.02 ± 0.19_bc_^cd^	3.76 ± 0.12_b_^de^	3.51 ± 0.25_b_^ef^	3.27 ± 0.42_b_^f^
	75E	5.00 ± 0.00_a_^a^	4.91 ± 0.19_b_^a^	4.79 ± 0.28_b_^a^	4.64 ± 0.35_b_^ab^	4.32 ± 0.33_bc_^bc^	4.14 ± 0.33_bc_^cd^	3.89 ± 0.17_bc_^cd^	3.91 ± 0.12_c_^d^
	100E	5.00 ± 0.00_a_^a^	4.94 ± 0.13_b_^ab^	4.88 ± 0.25_b_^a^	4.73 ± 0.16_b_^ab^	4.65 ± 0.31_c_^ab^	4.38 ± 0.27_c_^bc^	4.21 ± 0.41_c_^cd^	3.89 ± 0.15_c_^d^
Color	Control	5.00 ± 0.00_a_^a^	4.62 ± 0.24_a_^ab^	4.37 ± 0.19_a_^bc^	4.13 ± 0.15_a_^cd^	3.78 ± 0.23_a_^d^	2.33 ± 0.41_a_^e^	1.42 ± 0.28_a_^f^	1.10 ± 0.11_a_^f^
	0E	5.00 ± 0.00_a_^a^	4.81 ± 0.18_a_^ab^	4.59 ± 0.27_ab_^abc^	4.48 ± 0.15_ab_^bc^	4.21 ± 0.15_ab_^cd^	3.89 ± 0.19_b_^de^	3.58 ± 0.34_b_^ef^	3.26 ± 0.37_b_^f^
	50E	5.00 ± 0.00_a_^a^	4.85 ± 0.08_a_^ab^	4.70 ± 0.23_ab_^abc^	4.53 ± 0.26_b_^bc^	4.39 ± 0.27_b_^c^	3.98 ± 0.26_b_^d^	3.77 ± 0.28_b_^d^	3.22 ± 0.27_b_^e^
	75E	5.00 ± 0.00_a_^a^	4.89 ± 0.26_a_^ab^	4.82 ± 0.16_b_^ab^	4.51 ± 0.25_b_^bc^	4.28 ± 0.39_b_^c^	4.05 ± 0.26_b_^cd^	3.76 ± 0.14_b_^de^	3.49 ± 0.43_b_^e^
	100E	5.00 ± 0.00_a_^a^	4.92 ± 0.21_a_^a^	4.85 ± 0.14_b_^ab^	4.69 ± 0.15_b_^abc^	4.51 ± 0.25_b_^bc^	4.43 ± 0.32_b_^c^	4.36 ± 0.17_c_^c^	3.74 ± 0.23_b_^d^
Odor	Control	5.00 ± 0.00_a_^a^	4.53 ± 0.31_a_^b^	4.42 ± 0.12_a_^b^	4.21 ± 0.21_a_^b^	3.42 ± 0.30_a_^c^	2.47 ± 0.29_a_^d^	1.65 ± 0.16_a_^e^	1.14 ± 0.19_a_^f^
	0E	5.00 ± 0.00_a_^a^	4.78 ± 0.24_a_^ab^	4.55 ± 0.17_ab_^bc^	4.37 ± 0.37_a_^cd^	4.07 ± 0.37_b_^de^	3.91 ± 0.24_b_^ef^	3.52 ± 0.15_b_^fg^	3.35 ± 0.11_b_^g^
	50E	5.00 ± 0.00_a_^a^	4.85 ± 0.24_a_^ab^	4.69 ± 0.18_ab_^ab^	4.54 ± 0.22_a_^bc^	4.22 ± 0.13_b_^cd^	3.87 ± 0.12_b_^de^	3.83 ± 0.32_b_^ef^	3.48 ± 0.31_b_^f^
	75E	5.00 ± 0.00_a_^a^	4.86 ± 0.29_a_^ab^	4.60 ± 0.15_ab_^abc^	4.52 ± 0.41_a_^bc^	4.35 ± 0.45_b_^c^	4.21 ± 0.15_bc_^c^	3.57 ± 0.25_bc_^d^	3.39 ± 0.08_b_^d^
	100E	5.00 ± 0.00_a_^a^	4.88 ± 0.11_a_^ab^	4.72 ± 0.19_b_^abc^	4.58 ± 0.25_a_^bcd^	4.43 ± 0.16_b_^cd^	4.37 ± 0.15_c_^cd^	4.22 ± 0.33_c_^de^	3.91 ± 0.27_c_^e^
Overall acceptability	Control	5.00 ± 0.00_a_^a^	4.56 ± 0.21_a_^b^	4.31 ± 0.08_a_^bc^	4.08 ± 0.19_a_^c^	3.52 ± 0.17_a_^d^	2.78 ± 0.21_a_^e^	1.73 ± 0.12_a_^f^	1.27 ± 0.12_a_^g^
	0E	5.00 ± 0.00_a_^a^	4.70 ± 0.35_a_^ab^	4.57 ± 0.15_ab_^b^	4.38 ± 0.15_b_^bc^	4.15 ± 0.15_b_^cd^	3.93 ± 0.14_b_^de^	3.85 ± 0.40_b_^de^	3.61 ± 0.07_b_^e^
	50E	5.00 ± 0.00_a_^a^	4.79 ± 0.21_a_^ab^	4.68 ± 0.29_ab_^abc^	4.53 ± 0.19_b_^bc^	4.38 ± 0.31_b_^c^	3.89 ± 0.26_b_^d^	3.75 ± 0.27_b_^d^	3.58 ± 0.18_b_^d^
	75E	5.00 ± 0.00_a_^a^	4.85 ± 0.43_a_^ab^	4.77 ± 0.36_b_^ab^	4.45 ± 0.11_bc_^bc^	4.15 ± 0.28_bc_^c^	4.06 ± 0.14_b_^cd^	3.69 ± 0.12_b_^de^	3.53 ± 0.25_b_^e^
	100E	5.00 ± 0.00_a_^a^	4.91 ± 0.16_a_^a^	4.81 ± 0.27_b_^ab^	4.76 ± 0.10_c_^ab^	4.71 ± 0.18_c_^abc^	4.51 ± 0.39_c_^bc^	4.38 ± 0.14_c_^c^	3.79 ± 0.15_b_^d^

*Note*: The values are expressed as mean ± standard deviation (n = 8). 0E: 0% ethanol extract (water extract), 50E: 50% ethanol extract, 75E: 75% ethanol extract, and 100E: 100% ethanol extract. Subscript letters within the same column indicate significant (*p* > 0.05) differences of means within the treatments. Superscript letters within the same row indicate significant (*p* > 0.05) differences of means within the storage time.

## 4 CONCLUSIONS

Extension of shelf life using natural antioxidants like water lily extracts is more desirable for highly perishable seafood preservation than synthetic ones. In this study, water lily ethanolic extract (100E) has a considerable amount of antioxidant and antibacterial potentiality, which effectively reduced the quality deterioration, inhibited lipid oxidation, slowed down the microbial growth, and improved the overall sensory attributes in frozen Nile tilapia fillets. Based on the chemical, microbiological, and sensory data, it is suggested to use ethanolic extracts to enhance the quality and shelf life of Nile tilapia fillets during frozen storage. This preservation strategy can be applied in the fish/seafood processing industry and for household purposes as well.

## AUTHOR CONTRIBUTIONS

Md. Apon Dulal, Israt Jahan, and Md. Golam Rasul: Performed the experiments; analyzed and interpreted the data; and wrote the paper. Md. Rabiul Islam and Murshida Khan: Data curation and revised the manuscript. A. K. M. Azad Shah: Conceived and designed the study; contributed reagents/materials/analysis tools; and reviewing the original manuscript.

## CONFLICT OF INTEREST

The authors declare that there is no conflict of interest.

## DATA AVAILABILITY STATEMENT

Data are available upon request from the authors.
